# A mathematical model for predicting the spatiotemporal response of breast cancer cells treated with doxorubicin

**DOI:** 10.1080/15384047.2024.2321769

**Published:** 2024-02-27

**Authors:** Hugo J. M. Miniere, Ernesto A. B. F. Lima, Guillermo Lorenzo, David A. Hormuth II, Sophia Ty, Amy Brock, Thomas E. Yankeelov

**Affiliations:** a Department of Biomedical Engineering, The University of Texas at Austin, Austin, USA; b Oden Institute for Computational Engineering and Sciences, The University of Texas at Austin, Austin, USA; c Department of Civil Engineering and Architecture, University of Pavia, Lombardy, Italy; d Livestrong Cancer Institutes, The University of Texas at Austin, Austin, USA; e Department of Diagnostic Medicine, The University of Texas at Austin, Austin, USA; f Department of Oncology, The University of Texas at Austin, Austin, USA; g Division of Diagnostic Imaging, The University of Texas M.D. Anderson Cancer Center, Houston, USA

**Keywords:** Time-resolved microscopy, data assimilation, mechanism-based modeling, mathematical oncology

## Abstract

Tumor heterogeneity contributes significantly to chemoresistance, a leading cause of treatment failure. To better personalize therapies, it is essential to develop tools capable of identifying and predicting intra- and inter-tumor heterogeneities. Biology-inspired mathematical models are capable of attacking this problem, but tumor heterogeneity is often overlooked in *in-vivo* modeling studies, while phenotypic considerations capturing spatial dynamics are not typically included in *in-vitro* modeling studies. We present a data assimilation-prediction pipeline with a two-phenotype model that includes a spatiotemporal component to characterize and predict the evolution of *in-vitro* breast cancer cells and their heterogeneous response to chemotherapy. Our model assumes that the cells can be divided into two subpopulations: surviving cells unaffected by the treatment, and irreversibly damaged cells undergoing treatment-induced death. MCF7 breast cancer cells were previously cultivated in wells for up to 1000 hours, treated with various concentrations of doxorubicin and imaged with time-resolved microscopy to record spatiotemporally-resolved cell count data. Images were used to generate cell density maps. Treatment response predictions were initialized by a training set and updated by weekly measurements. Our mathematical model successfully calibrated the spatiotemporal cell growth dynamics, achieving median [range] concordance correlation coefficients of > .99 [.88, >.99] and .73 [.58, .85] across the whole well and individual pixels, respectively. Our proposed data assimilation-prediction approach achieved values of .97 [.44, >.99] and .69 [.35, .79] for the whole well and individual pixels, respectively. Thus, our model can capture and predict the spatiotemporal dynamics of MCF7 cells treated with doxorubicin in an *in-vitro* setting.

## Introduction

Chemoresistance, the ability of cancer cells to evade or endure the cytotoxic effects of drugs and therapies, is a fundamental contributing factor to treatment failure and is associated with poor prognosis, disease recurrence, and metastasis in patients.^1,2^ Driven by a variety of phenomena such as phenotypic resistance (i.e., phenotypic traits blocking drugs’ mechanisms of action), altered metabolism, or genetic changes caused by the treatment itself,^3–6^ a key component of chemoresistance and tumor relapse following therapy lies in the heterogeneous genetic and phenotypic structure of solid neoplasms, allowing regrowth from even a small number of resistant cells.^4,7–9^

Both genetic and non-genetic factors contribute to the development of intra- and intertumoral heterogeneity. Among the latter, the degree to which stromal cells infiltrate the microenvironment as well as how new vasculature is recruited to the tumor may result in a heterogeneous delivery of both nutrients and treatments. This heterogeneity can lead to the formation of habitats; i.e., subregions within the tumor that are distinguished by unique phenotypic signatures and properties.^10–12^ The spatially varying habitats contribute to varied treatment response across the tumor as the local environment can affect both the delivery and efficacy of the selected treatment.^13^ Thus, the design of treatment protocols accounting for these intra- and inter-tumoral variations would enable the optimal improvement of therapeutic outcomes for each individual patient. To achieve this goal, it is fundamental to leverage a rigorous methodology that explicitly accounts for spatiotemporal heterogeneities in individual tumors.^14^

Mathematical models that account for the underlying biology of the spatial and temporal development of tumors – as well as their response to therapy – have been shown to accurately predict the response of individual patient tumors.^15–17^ In particular, there has been much progress in using imaging-based measurements to calibrate and constrain model predictions in both the pre-clinical and clinical settings.^18–28^ Importantly, it is difficult to develop and validate biology-based mathematical models in the clinical setting where there are fundamental limitations to the amount and quality of imaging data that can be acquired on an individual patient. Furthermore, models that account for different cell types that can be calibrated to available data are rare^29–31^; and although studies employing compartmental models have been developed over the past decade,^32–34^ phenotypic heterogeneities are not typically incorporated into mathematical modeling studies of clinical data. Models that seek to capture the dynamics of multiple interacting phenotypes are typically investigated in *in vitro* or *in silico* settings. Furthermore, such models typically disregard the spatial dynamics and heterogeneity at play in cells cultivated *in vitro*; rather, they emphasize characterizing the average cell population growth observed across the whole assay.^35^

Time-resolved microscopy is a high-resolution imaging technique that captures images of biological samples at specific intervals over extended durations with minimal intervention. It allows frequent, noninvasive, cell-preserving imaging, and therefore does not suffer from the data scarcity issues of clinical studies. For example, A coupled model of pharmacokinetics and pharmacodynamics was created and validated by McKenna *et al*. and was able to forecast the reaction of a uniform cell population to a treatment schedule that changed over time. The researchers used fluorescence microscopy over a period of time to examine the absorption of doxorubicin and how the cell population responded to this medication across a range of triple negative breast cancer cell lines.^36^ Tyson *et al*. calibrated a model using immunofluorescence labeling and time-lapse automated microscopy, allowing them to estimate the lifespan of individual lung cancer cells.^37^ Lima *et al*. used time-resolved microscopy with a phase-field tumor growth model to calibrate rates of proliferation, apoptosis, necrosis and mobility on *in vitro* human liver carcinoma cell subpopulations.^38^ Additionally, by raster scanning an entire well-plate, dynamic spatial information on cell distribution can also be obtained over time. Most relevant to our work, Howard *et al*. employed time-resolved microscopy to image several breast cancer cell lines treated with various doses and regimens of doxorubicin, and then developed a family of mathematical models to characterize their growth and response to therapy.^39^ Expanding on this approach, Yang *et al*. constructed a set of ordinary differential equations that were able to recapitulate each treatment regimen regardless of the concentration or number of doxorubicin doses.^18^ In particular, Yang *et al*. modeled *in vitro* breast cancer colonies treated with doxorubicin as compartmentalized subpopulations of resistant and irreversibly damaged cells, allowing for phenotypic switching of cells in response to the application of an additional dose. While successfully capturing the temporal evolution of MCF7 cancer cells and their response to a variety of treatment regimens, their study did not include spatial considerations of cell distribution. Moreover, they did not establish a pipeline allowing for the prediction of growth and treatment response, a key step of crucial clinical importance.

In this contribution, we seek to broaden the work of Yang *et al*. in two directions. First, we incorporate a spatial component to their modeling framework that aims at explicitly capturing heterogeneities in growth and response to treatment in terms of tumor cell density. Second, we develop a computational pipeline to recapitulate and predict of the spatiotemporal heterogeneous dynamics of tumor cell density with our model. Toward this end, we leverage the data assimilation methodology proposed by Liu *et al*. to provide model updates as more data becomes available from time-resolved microscopy measurements.^40^ Then, we further demonstrate that our spatiotemporal modeling approach can recapitulate and forecast tumor cell density dynamics in a two-dimensional *in vitro* setting by leveraging the same dataset of MCF7 cells treated with doxorubicin used by Yang *et al*. Once a mathematical model has been shown to accurately predict the spatial and temporal development of a tumor, it can potentially be used to predict and optimize therapeutic responses. If the model is accurate, then one can simulate a range of interventions and select the one that provides the greatest tumor control.^41–43^ For example, in the present case, one could use our pipeline to identify a doxorubicin treatment schedule to maintain tumor control for as long as possible.

The remainder of this study is organized as follows. We first summarize the experimental methods and derivation of the model introduced by Yang *et al*.^18^ We then describe the model calibration approach, how the initial predictions are made, and how we update our forecasts during the experiment. We conclude with a discussion of the significance of the study, its limitations, and possible directions for future investigation.

## Materials and methods

### Cell culture and experimental conditions

As the data employed in this study have been previously published, we highlight only the salient details. (For a complete description of the experimental methods, please see ref. 39) ER-positive MCF7 breast cancer cells (ATCC HTB-22) were grown in a Minimum Essential Media (Gibco)/10% fetal bovine serum (Gibco)/1% penicillin-streptomycin (Gibco) mix and maintained at 37°C in a 5% CO2 incubator. Cells were genetically modified to express EGFP (enhanced green fluorescence protein) along with a nuclear localization signal (MCF7-EGFPNLS1) integrated using the Sleeping Beauty transposon system. The EGFP-NLS sequence, obtained as a gBlock (IDT), was cloned into the optimized Sleeping Beauty transfer vector pSBbi-Neo (gifted by Eric Kowarz, Addgene plasmid #60525).^44^ Finally, the two-plasmid system was transfected into the cell population using Lipofectamine 2000.^45^ EGFP-expressing cells were then collected through fluorescence activated cell sorting and cultured in G418 (Caisson Labs)-supplemented media (200 ng/ml).

Cells were then seeded onto a 96-well plate at 2000 cells per well and allowed to grow untreated in 100 µl of culture media for 48 hours. Next, 100 µl of media containing doxorubicin hydrochloride (Cayman Chemical 15,007, Ann Arbor, Michigan) at 2× the desired final concentration was added into each well for a 24-hour duration and then washed with fresh growth media containing no drug. Nine different doxorubicin concentrations were investigated: 10, 20, 35, 50, 75, 100, 125, 150, and 300 nM with *n* = 6 replicates per concentration, along with six untreated replicates as a control.

### Data gathering and image processing

**Figure 1.**
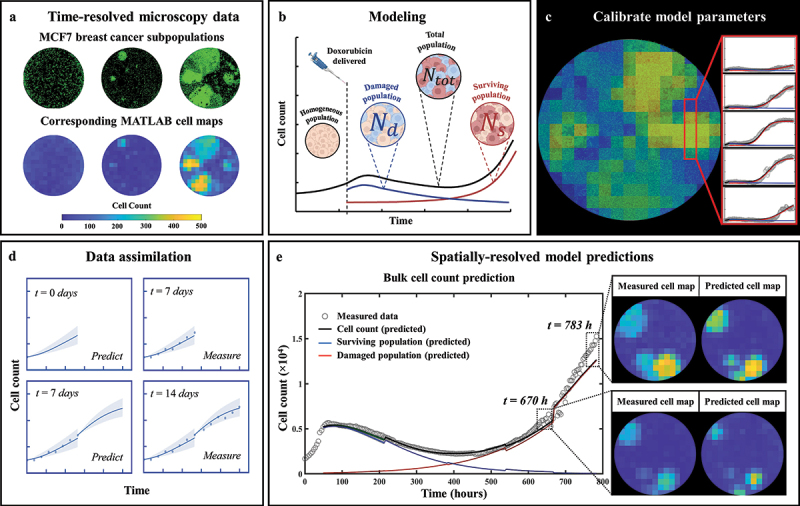
General flowchart of predicting MCF7 growth through data assimilation. (a) MCF7 breast cancer cells are grown and treated with varying doses of doxorubicin. Fluorescent microscopy is used to gather images every four hours which are converted into cell density maps. (b) A two-subpopulation model is used to describe the spatial and temporal development of MCF7 cells. (c) The model is calibrated on the cell density maps, yielding parameter values for each individual pixel. (d) We use a data assimilation pipeline consisting of successive short-term predictions guided by serial measurements. e. Cell density maps and bulk cell counts (i.e., total cell count across the entire well) are predicted at weekly intervals.

Fluorescent and phase contrast images (Figure 1(a)) were collected every 2–4 hours using an IncuCyte S2 Live Cell Analysis System (Essen/Sartorius, Goettingen, Germany) for 1000 hours. Cell quantification and centroid positions were gathered through background subtraction, thresholding, edge detection and minimum area filtering. The resulting time courses of cell count were truncated once confluence was reached.^18^ Cell centroid positions were used to generate 2D tumor cell density maps (see Figure 1(a)), which are then used to calibrate the mathematical model described in the next section. The resolution of the cell maps was limited by signal-to-noise considerations and chosen to be 430 µm × 430 µm, corresponding to a grid size of 15 × 15 pixels over a 6450 µm × 6450 µm field-of-view of each well.

### Mathematical model

The model used in this study corresponds to a two-dimensional reaction-diffusion extension of the single-dose model presented by Yang *et al*. (Figure 1(b)).^18,46^ Such models are routinely used in cancer modeling. Hence, our model formulation accounts for cell migration and characterizes spatial heterogeneities arising both in the pre- and post-treatment phase of the experiment.

Prior to exposure to doxorubicin, the tumor cell population is assumed to be phenotypically homogeneous, expanding by diffusion, and growing freely according to exponential growth:

(1) $$\frac{\partial N_{\mathrm{tot}}\left(\overset{ˉ}{x},t\right)}{\mathrm{\partial t}}=\nabla .\left(D\left(\overset{ˉ}{x}\right)\nabla N_{\mathrm{tot}}\left(\overset{ˉ}{x},t\right)\right)+g\left(\overset{ˉ}{x}\right)N_{\mathrm{tot}}\left(\overset{ˉ}{x},t\right)$$

(2) $$N_{\mathrm{tot}}\left(\overset{ˉ}{x},0\right)=N_{0}\left(\overset{ˉ}{x}\right),$$

where $N_{\mathrm{tot}}\left(\overset{ˉ}{x},t\right)$ is the total tumor cell count at location $\overset{ˉ}{x}$and time t, and *D*($\overset{ˉ}{x}$) and *g*($\overset{ˉ}{x}$) are the spatially defined diffusion coefficient and proliferation rate of the untreated cells, respectively. To reflect the homogeneous nature of the cell population in the absence of treatment, we consider that the cells diffuse and grow independent of their position $\overset{ˉ}{x}$; thus, we set *D*($\overset{ˉ}{x}$) ≡ *D*_*0*_, and *g*($\overset{ˉ}{x}$) ≡ *g*_*0*_.

After doxorubicin treatment is applied at time *t*_*dox*_ *=* 48 hours, the previously homogeneous cell population (as described by Equations (1) and (2)) is now modeled as two subpopulations distinguishable by their reaction to the drug: a “surviving” subpopulation, with cell count $N_{s}\left(\overset{ˉ}{x},t\right)$, that will evade doxorubicin-induced death, and an “irreversibly damaged” subpopulation, with cell count $N_{d}\left(\overset{ˉ}{x},t\right)$, that will eventually die after a short period of growth. The fractions of the surviving and irreversibly damaged subpopulations at time *t = t*_*dox*_ are defined as *f*_*s*_ and (1 – *f*_*s*_), respectively.

Thus, we have:

(3) $$N_{s}\left(\overset{ˉ}{x},t_{\mathrm{dox}}\right)=f_{s}\left(\overset{ˉ}{x}\right)N_{\mathrm{tot}}\left(\overset{ˉ}{x},t_{\mathrm{dox}}\right),$$

(4) $$N_{d}\left(\overset{ˉ}{x},t_{\mathrm{dox}}\right)=\left(1-f_{s}\left(\overset{ˉ}{x}\right)\right)N_{\mathrm{tot}}\left(\overset{ˉ}{x},t_{\mathrm{dox}}\right),$$

with the total cell count in the post-treatment phase given by:

(5) $$N_{\mathrm{tot}}\left(\overset{ˉ}{x},t\right)=N_{s}\left(\overset{ˉ}{x},t\right)+N_{d}\left(\overset{ˉ}{x},t\right).$$

The surviving subpopulation is assumed to follow logistic growth within a reaction-diffusion equation:

(6) $$\frac{\partial N_{s}\left(\overset{ˉ}{x},t\right)}{\mathrm{\partial t}}=\nabla \cdot \left(D_{s}\left(\overset{ˉ}{x}\right)\nabla N_{s}\left(\overset{ˉ}{x},t\right)\right)+g_{s}\left(\overset{ˉ}{x}\right)\left(1-\frac{N_{\mathrm{tot}}\left(\overset{ˉ}{x},t\right)}{\theta }\right),$$

where $g_{s}\left(\overset{ˉ}{x}\right)$is the post-treatment proliferation rate, $D_{s}\left(\overset{ˉ}{x}\right)$ the diffusion coefficient of surviving cells (where we again assumed that the cells diffuse independent of their position, i.e., $D_{s}\left(\overset{ˉ}{x}\right)=D_{s}$), and θ the carrying capacity (i.e., the maximum admissible cell density). The irreversibly damaged subpopulation is assumed to initially follow logistic growth, which progressively transitions into treatment-induced death as continued drug uptake triggers DNA damage and cell cycle disruption leads to cell death^36,47–49^:

(7) $$\begin{array}{r} \frac{\partial N_{d}\left(\overset{ˉ}{x},t\right)}{\mathrm{\partial t}} \end{array}=\nabla \cdot \left(D_{d}\left(\overset{ˉ}{x}\right)\nabla N_{d}\left(\overset{ˉ}{x},t\right)\right)\;\;\;\;\text{\textbackslash{}break}\;\;\;\;\;\;\;+\left(\left(g_{d}\left(\overset{ˉ}{x}\right)+k_{d}\left(\overset{ˉ}{x}\right)\right)\exp\left(-\gamma_{d}\left(\overset{ˉ}{x}\right)\left(t-t_{\mathrm{dox}}\right)\right)\text{\textbackslash{}break}\;\;\;-k_{d}\left(\overset{ˉ}{x}\right)\right)N_{d}\left(\overset{ˉ}{x},t\right)\left(1-\frac{N_{\mathrm{tot}}\left(\overset{ˉ}{x},t\right)}{\theta }\right),$$

where $g_{d}\left(\overset{ˉ}{x}\right)$ and $k_{d}\left(\overset{ˉ}{x}\right)$ respectively represent the proliferation and doxorubicin-induced death rates, $\gamma_{d}\left(\overset{ˉ}{x}\right)$ denotes the doxorubicin-induced death delay rate (which characterizes the time it takes for the cells to transition from proliferating to dying following drug exposure), $D_{d}\left(\overset{ˉ}{x}\right)$ is the diffusion coefficient of the damaged cells (also assumed independent of position; i.e., $D_{d}\left(\overset{ˉ}{x}\right)=D_{d}$), and *θ* is the same carrying capacity as in Equation (6). Note that the pre-treatment population followed exponential growth (Equation (1)) while the post-treatment subpopulations are modeled using logistic growth (Equations (6) and (7)). This is explained by the low cell density in the pre-treatment phase, allowing a simplification from a logistic to an exponential form. As the cell count is susceptible to rise sharply with cells reaching maximum capacity in the post-treatment phase, a logistic model is now used.

Moreover, for simplicity, we assume that the untreated, surviving, and irreversibly damaged cells diffuse similarly regardless of their treatment status i.e., *D*_*s*_ *= D*_*d*_ *= D*_*0*_. As such, diffusion is calibrated in pre-treatment phase only, and is then fixed post-treatment. Figure 1(b) presents a graphical representation of the model compartments during the two experiment phases, and Table 1 summarizes the model parameters. The partial differential equations were solved using the finite difference method over the pixel grid, with fully explicit time differentiation (time step Δt = 1 hr), along with central differences for spatial discretization and no flux boundary conditions.

**Table 1.** List of model parameters.

Parameter	Definition	Notes
*g0*	Proliferation rate of untreated cells (hr^−1^)	Global, calibrated in pre-treatment phase (range: [.02 .035])
*D0*	Diffusion coefficient (mm^2^/hr^−1^)	Global, calibrated in pre-treatment phase (range: [0.005])
*gs*	Proliferation rate of surviving cells (hr^−1^)	Local, calibrated (range: [.0001 .035])
*fs*	Surviving cell fraction at time of treatment	Global, calibrated (range: [0 1])
*kd*	Drug-induced death rate of irreversibly-damaged cells (hr^−1^)	Global, calibrated (range: [.0001 .01])
*gd*	Proliferation rate of irreversibly damaged cells (hr^−1^)	Global, calibrated (range: [.001 .05])
*γd*	Drug-induced death delay rate (hr^−1^)	Global, calibrated (range: [1/120 1/10])
*θ*	Carrying capacity	Global, calibrated within a narrow range (range: [88000 90,000])

### Global and local parameters

The model presented in Equations (1), (6), and (7) contains eight parameters (i.e. *g*_*0*_, *D*_*0*_, *g*_*s*_, *f*_*s*_, *g*_*d*_, *k*_*d*_, *γ*_*d*,_ and *θ)*. Given the number of pixels per cell map (see ‘*Data gathering and image processing’* section above), one parameter value per pixel for each of the eight parameters would require calibrating several hundred parameters and almost certainly lead to overfitting. To avoid this issue, a global sensitivity analysis was carried out to determine the contribution of each model parameter on the spatiotemporal dynamics of tumor cells. This was performed by setting all parameters global and randomly sampling values from their respective parameter spaces (see Table 1) to generate 5000 model evaluations and calculate the total effect index for each parameter. The results (see Figure S1 of the Supplementary Materials) indicated that the two parameters having the greatest effect on the tumor cell density (i.e., small changes in their values leading to the greatest changes on the cell number) were the proliferation rate of the surviving subpopulation, *g*_*s*_ and the carrying capacity, *θ*. We therefore decided to calibrate all other parameters in a global fashion, meaning that a single parameter value will be calibrated over the whole well and shared by all pixels. Moreover, the carrying capacity *θ* is assumed to be a physical parameter characterizing the experimental conditions (e.g., type of cell, well dimensions), which may only exhibit narrow variations emanating from diversity in seeding, dosing, and ensuing growth. Thus, the carrying capacity *θ* was also calibrated globally over a narrow range of admissible values. The surviving growth rate *g*_*s*_ is thus the only parameter with a different value in each pixel. As will be shown in the ‘Results’ section below, this modeling choice suffices to capture the experimentally observed spatial heterogeneity.

### Model fitting

We fit our model to each replicate from the time-resolved data obtained as described above in the ‘*Cell culture and experimental conditions*’ section. Model calibration (Figure 1(c)) was performed using a trust-region reflective algorithm via MATLAB’s (R2021a, The Mathworks, Natick, MA) nonlinear least-squares fitting function, *lsqnonlin*. Parameter bounds and initial guesses were inspired by ref. 18. Calibration precision is evaluated using a bootstrapping approach (100 bootstrap samples). This is performed by calibrating the model to the data (yielding a first parameter set), and using the calibration residual as an estimate of the normally distributed noise. A new synthetic dataset can now be created by sampling a new residual from the noise distribution and adding it to the model calibration. The model can now be calibrated onto the synthetic dataset, yielding another parameter set. This process is repeated 100 times, providing normal distributions for each parameter corresponding to the calibration precision as quantified by calculating the interquartile range.

### Prediction scheme

Predictions of the spatiotemporal tumor cell dynamics were performed through a leave-one-out approach employing a data assimilation pipeline inspired by Liu *et al*. ^37^ (Figure 1(d,e)). In brief, with this method we seek to make short-term predictions which are then updated at regular intervals as more data becomes available for the replicate whose dynamics we are predicting. As we are most interested in accounting for spatial heterogeneities, and these are not observed prior to treatment (i.e., nearly no inter- or intra-well heterogeneity), the pre-treatment phase is excluded from our prediction approach; that is, our predictions begin at the time the drug is applied. We next describe the details of each step in our data assimilation-prediction approach.

**Figure 2.**
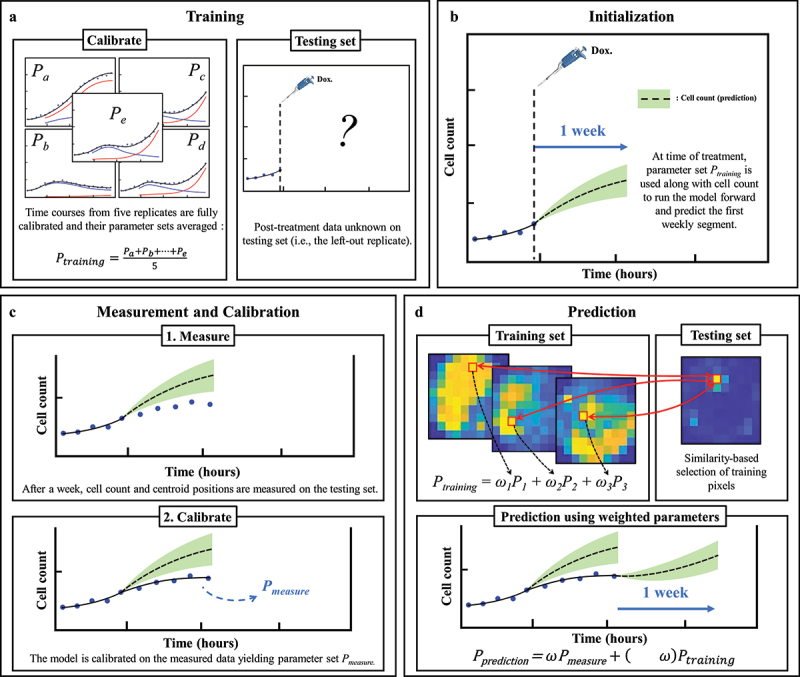
Predicting MCF7 cell growth and response to doxorubicin through leave-one-out data assimilation-prediction pipeline. (a) Out of six replicates treated with similar doxorubicin doses, five are selected to form the training set and fully calibrated. Their parameter sets are averaged into parameter set *P*_*training*._ the post-treatment data on the left-out replicate are *a priori* “hidden”. (b) for the replicate in the testing set, the data at the time of the treatment are used to run the model forward with parameter set *P*_*training*,_ thereby yielding a first prediction. (c) After one week, the model is informed by the data collected during this timeframe and calibrated accordingly, thereby yielding parameter set *P*_*measure*_. (d) to predict tumor cell dynamics over the subsequent week, we first estimate the new values of the model parameters based on the degree of similarity between the tumor cell dynamics in each pixel of the replicate in the testing set and all the pixels in the training set. This comparison yields *P*_*training*_, which we combine with *P*_*measure*_ to obtain the estimated parameter values for model prediction (i.e., *P*_*prediction*_). The green shaded regions in panels b-d represent the bootstrap interquartile range of the predictions, around the dashed line representing the bootstrapped mean prediction.

For the *n* = 6 replicates receiving the same dose of doxorubicin, a training set of *n*_*training*_ = 5 replicates is selected, and the model is calibrated to each of these five datasets using the whole time course of measurements collected during the experiment. The five resulting parameter sets are then averaged into a parameter set *P*_*training*_ (15 × 15 × 6 matrix, corresponding to the grid size and number of post-treatment model parameters). The remaining replicate is placed in the testing set (*n*_*testing*_ = 1). For this replicate, the prediction of the dynamics of the tumor cell population follows a series of subsequent data assimilation-prediction steps over time periods of one week. During the first week after treatment, we run the model forward using the cell count of that replicate at the time the treatment is applied as initial conditions and leveraging the parameter set *P*_*training*_ (Figure 2(a,b)). The first week of post-treatment data is then measured for the replicate, allowing calibration of Equation (6)–(7) and yielding parameter set *P*_*measure*_ (Figure 2(c)). An individualized prediction over the subsequent week initialized using the last time point measured can now be performed on each pixel through the following three steps. First, for each pixel *p* within the testing set, the 5 × 15 × 15 pixels in the training set are ranked based on the similarity of their time course of cell number to the time course observed in *p* (from the time of the treatment to the last available time point in *p*). The similarity measure is the concordance correlation coefficient (*CCC*).^50^ Second, the model parameters associated with the pixels in the training set with the 25% most similar time courses to that of pixel *p* in the testing set are averaged (in a weighted fashion) to form the parameter set *P*_*training,p*_ according to their degree of similarity:

(8) $$P_{\mathrm{training},p}=\overset{N}{\underset{k=1}{\sum }}\alpha_{k}P_{k},$$

where *N* = 282 (i.e., .25 × 5 × 15 × 15), *P*_*k*_ the calibrated parameter set of pixel *k* from the training set, and $\alpha_{k}$ the weight assigned to this pixel. The weights $\alpha_{k}$ are determined by dividing the *CCC* of the time course of pixel *k* by the sum of the *CCC*s of all 282 pixels, that is:

(9) $$\alpha_{k}=\frac{CCC_{k}}{\sum_{j=1}^{N}\mathrm{CC}C_{j}}.$$

Note that, as per Eq. (8), all the six post-treatment parameters used for the predictions can now vary pixel-wise, as opposed to the calibrated parameters of which only one (surviving proliferation rate *g*_*s*_) varied pixelwise. This was necessary during the calibration step for reasons of overfitting mentioned above, but is no longer necessary when predicting weekly segments as this step only requires forward model evaluations. Third, *P*_*training*_ (now a new 15 × 15 × 6 matrix containing all *P*_*training,p*_ from Eq. (8)) is weighted against *P*_*measure*_ to yield the final parameter set, *P*_*prediction*_, allowing the prediction of the dynamics of the tumor cell population over the following week (Figure 2(d)):

(10) $$P_{\mathrm{prediction}}=\omega P_{\mathrm{training}}+\left(1-\omega \right)P_{\mathrm{measure}}.$$

As time progresses and more data is acquired for the test dataset, *ω* decreases to give more weight to the calibrated parameter set, *P*_*measure*_, which is specific to the replicate for which we are obtaining predictions of tumor cell dynamics. Inspired by ref. 37, we use:

(11) $$\omega =0.6{\hat{t}}^{2}-1.2\hat{t}+1.0$$

with $\hat{t}$ the time in hours divided by the time at which the final time point is gathered on the longest experiment (963 hours) so that $\hat{t}$ ranges from 0 to 1. The form of *ω* allows *ω*(0) = 1 giving greater weight to the training set on the early time points, followed by a decrease shifting the weight to the individual replicate’s parameter set. The quadratic form was chosen because it decreases slower than a linear function during the second half of the experiment and is virtually unchanged in the neighborhood of the terminal timepoint (i.e., $\hat{t}$ = 1), thus maintaining the weight of the training set approximately constant on the final time points. The three steps described above are then repeated each week until reaching the end of the experiment. Notice that the surviving fraction, *fs* (previously defined as the fraction of surviving cells at time *t = t*_*dox*_), is also updated through the prediction pipeline and is now used to split the cells at each weekly timepoint.

Note that, as we use bootstrapping to evaluate the precision of our model calibrations in the replicates in the training set and in each weekly calibration for the replicate in the testing set, we can obtain a distribution of values for each model parameter in *P*_*training*_ and *P*_*measure*_. Thus, combining these parameter distributions via Equation 10, we can also obtain the corresponding distributions in *P*_*prediction*_. Then, we can sample the parameter distributions for *P*_*prediction*_ and quantify the precision of our model predictions. Toward this end, we randomly draw 1000 samples and obtain an equivalent number of predictions of tumor cell dynamics during each week of the data assimilation-prediction pipeline as outlined above. In particular, we focus on analyzing the mean and interquartile range of the predicted tumor cell counts in each pixel of the replicate in the testing set.

### Statistical analysis

To quantify the quality of the model calibrations and predictions we use the *CCC* (concordance correlation coefficient).^47^ This quantity measures the agreement between the model calibration or prediction results and matching experimental data. We employ *CCC* to assess model performance in recapitulating and predicting tumor cell counts both at global and local level. Hence, we denote by *CCC*_*well*_ the use of this metric to compare the measured time course of the total cell count of the entire well to that obtained from the model calibration or prediction. Similarly, we denote by *CCC*_*pixel*_ the use of this metric to measure the quality of the local, pixel-wise calibration or prediction by calculating the average over the well of the *CCC*s that compare each pixel’s time course of total cell count to that obtained from model calibration or prediction.

## Results

### Fluorescent microscopy of MCF7 cells

**Figure 3.**
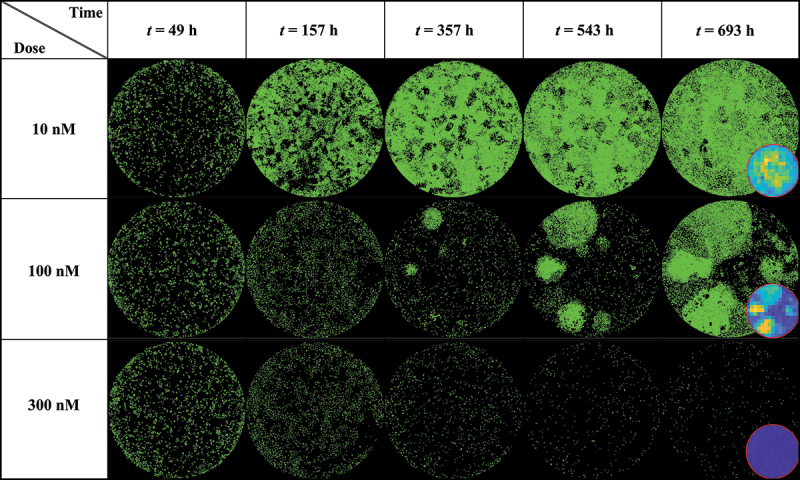
MCF7 breast cancer cells at various time points and treated with 10, 100 and 300 nM of Doxorubicin. Data show that low (10 nM) and high (300 nM) doses do not generate significant spatial heterogeneity post treatment, as opposed to a medium dosage where clusters of highly proliferating cells are responsible for uneven cell distribution after approximately two weeks of growth. Corresponding MATLAB tumor cell density maps can be seen on the last column.

Figure 3 shows a dish treated at low (10 nM), medium (100 nM), and high (300 nM) doses of doxorubicin at various time points (49, 157, 357, 543 and 693 hours after seeding), exhibiting clear patterns of spatial heterogeneity at a medium dosage. At a low dosage (10 nM, top row), cell growth is uniform across the well leading to cells covering the entirety of the dish without any distinctive pattern of spatial distribution. Thus, the effect of the treatment is negligible. At a high dosage (300 nM, bottom row), the powerful effect of the chemotherapy prevents any cell growth or recovery and the entire population homogeneously dies over the course of weeks. When a medium dosage (100 nM, middle row) is applied, rapid cell recovery is observed in localized areas forming clusters of highly proliferative cells responsible for uneven distribution. This intra-tumor spatial heterogeneity then translates into temporal inter-replicate heterogeneity, with each replicate showing distinct cell growth distribution patterns that lead to a diverse set of longitudinal time courses (see Supplemental Figure S2 from ref. 18 for complete dataset).

### Fitting phenotypic model to MCF7 cells treated with doxorubicin

**Figure 4.**
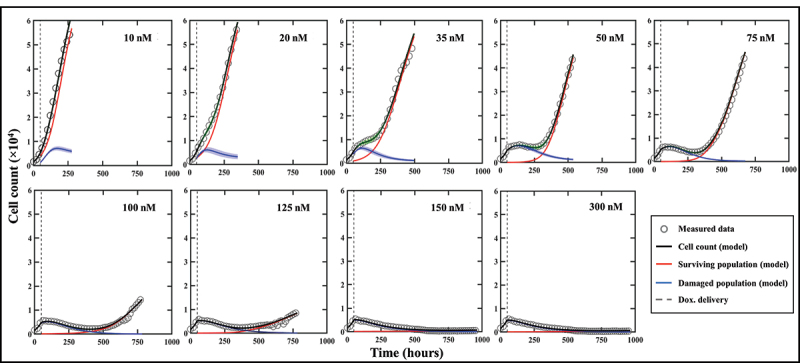
Model calibrations for representative replicates of MCF7 cells treated with varying doses of doxorubicin after 48 hours of growth. Measured bulk cell counts (gray circles) are shown along with the model’s estimates of the surviving (red), damaged (blue) and total (black) populations and their respective precisions. For clearer visualization, only one out of four data points are shown. The vertical dashed line represents the time of delivery of doxorubicin.

**Figure 5.**
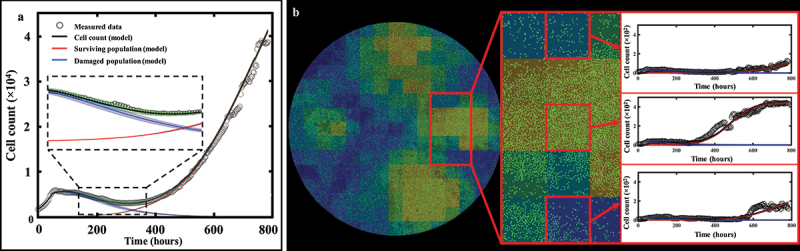
Spatial calibration of the two-subpopulation model on a replicate treated with 100 nM of doxorubicin. a. Bulk cell counts obtained for the global calibration of the model to the whole time course of microscopy measurements collected for the replicate, including the model’s estimate of the surviving, damaged, and total population cell counts. The calibration precision is shown on all three populations *via* the shaded areas. b. Our model is calibrated to fit the time course on each pixel of the well, thereby capturing the spatial heterogeneity of the tumor cell population dynamics. The cell density map is shown superimposed on the corresponding cell microscopy image, taken at the final time point (*t* = 782 h). The plots on the right illustrate the local counts of total, surviving, and damaged cells for three representative pixels exhibiting different temporal dynamics.

Figure 4 shows the tumor cell counts for representative replicates for each dose along with their associated model fit, demonstrating the ability of our spatial calibration to accurately characterize the dynamics of the bulk cell count (i.e., total cell count across the entire well) of treated MCF7 subpopulations. The calibration is performed for the whole duration of the experiment, and provides the model’s estimate of total, surviving, and damaged tumor cell counts along with their respective precisions displayed as shaded areas showing greater calibration uncertainty on the damaged population’s estimate. The quality of fit for the well and the pixels resulted in median and range *CCC*_*well*_ >.99 [.88, >.99] and *CCC*_*pixel*_ = .73 [.58, .85], respectively. Moreover, Figure 5(a) displays details of a local pixel calibration on a replicate treated with 100 nM of doxorubicin. Panel A shows the bulk cell counts obtained from the global fit, with *CCC*_*well*_ = .99. On panel B, the pixel map is superimposed on the fluorescence image at time *t* = 782 hours. Examples of calibrated pixels are shown, reflecting the model’s ability handle a wide variety of temporal dynamics and capture the elevated heterogeneity found within these *in vitro* tumor cell populations (*CCC*_*pixel*_ = .69) with only a single parameter calibrated locally (i.e., *g*_*s*_). The upper pixel exhibits a decay in cell count for approximately 500 hours before a slow cell recovery, contrasting with the middle pixel where a sharp proliferation phase begins around *t* = 300 hours. Lastly, the bottom pixel exhibits more unique dynamics, where a long decay of approximately 600 hours is followed by a sudden and brief increase in cell count, indicating a cell infiltration from neighboring pixels. As will be discussed below, such phenomena are poorly captured by the modeling approach.

**Figure 6.**
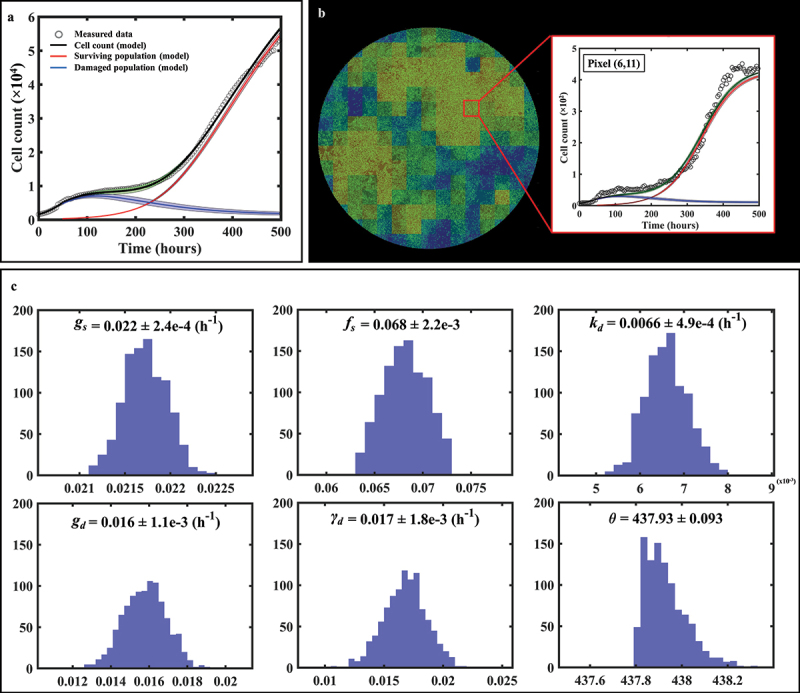
Spatial calibration of the two-phenotype model on a replicate treated with 35 nM of doxorubicin. (a) Bulk model calibration providing the model’s estimate of the surviving, damaged, and total population. The calibration precision is shown on all three populations (b) Cell map at final time point along with model calibration on an individual pixel at final time point (*t* = 497 h). (c) Distributions of each six post-treatment model parameters for pixel (6,11). The largest spreads are found on *k*_*d*_, *g*_*d*_ and *γ*_*d*_, indicative of a greater uncertainty on the estimate of the damaged population as reflected by its larger shaded area on panel a.

Figure 6 provides an example of the bootstrap parameter distributions resulting from global model calibration in a representative replicate treated with 35 nM of doxorubicin. Panel A shows the time-resolved cell counts of the replicate, along with the corresponding fit obtained from the global model calibration. The result of model calibration on an individual pixel is shown on panel B with the associated bootstrapped parameter distributions on panel 6C. Note that, as all parameters are globally calibrated with the exception of *g*_*s*_, the distributions for *f*_*s*_, *k*_*d*_, *g*_*d*_, *γ*_*d*_ and *θ* are the same for all the pixels of each individual replicate. The normalized standard deviations (standard deviations divided by the means, referred to as NSD) for those parameters indicate that the parameters with the largest spread are *k*_*d*_ (NSD = .074), *g*_*d*_ (NSD = .069) and _*d*_ (NSD = .11); that is, the parameters describing the damaged population and thus the effects of the treatment (NSDs for *g*_*s*_, *f*_*s*_ and *θ* are respectively .011, .032 and .00021). This observation can be made across all the replicates and doses, and it is aligned with the larger bootstrap interquartile range of the damaged populations depicted on Figure 4.

### Predictions of spatial tumor dynamics

**Figure 7.**
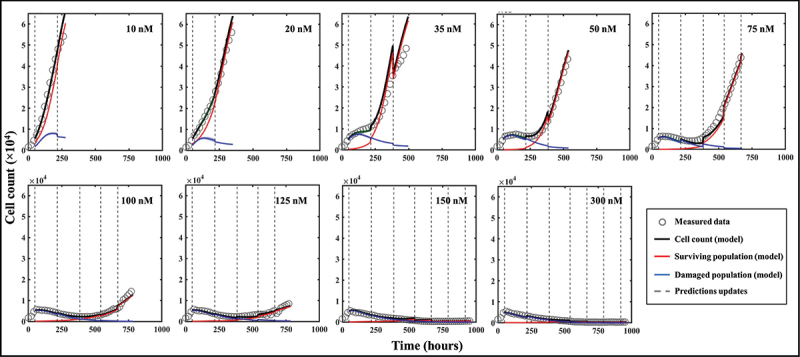
Model predictions of bulk cell count on representative replicates of MCF7 subpopulations treated with varying doses of doxorubicin after 48 hours of growth. Measured bulk cell count (gray circles) is shown along with the model’s predictions of the surviving (red), damaged (blue) and total (black) populations as well as their respective precisions. The predictions are performed on weekly segments indicated by the gray dashed lines. Our prediction pipeline is able to successfully forecast the total cell count of treated MCF7 colonies on weekly segments. For clearer visualization, only one out of four data points are shown.

**Figure 8.**
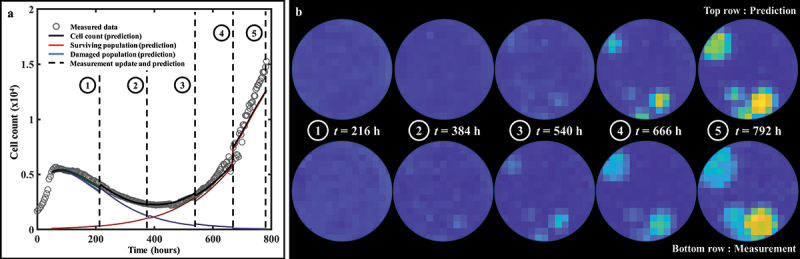
Spatiotemporal prediction of cell dynamics on a replicate treated with 100 nM of doxorubicin. (a) Prediction of bulk cell count. The prediction is performed on weekly segments and updated by measurements indicated by the vertical black dashed lines. (b) Comparisons between our model’s predictions of cell distribution and the map measured through microscopy at the time points indicated by the vertical dashed lines in panel A. The main patterns of heterogeneous cell distribution following doxorubicin treatment are successfully captured through data assimilation.

Representative predictions of total cell counts obtained through our data assimilation-prediction pipeline are shown in Figure 7. Again, predictions of total, surviving, and irreversibly damaged cell counts are shown along with the measured data. As outlined in the Methods section, the first prediction (week 0 to 1) is performed using the population parameter set from the training set, and predictions from week 1 to week 2 are then performed by calibrating the model from week 0 to week 1 and running the model forward in time from week 1 to 2. Then, predictions from week 2 to week 3 are performed by calibrating the model from week 0 to week 2, and running the model forward from week 2 to 3. This process is repeated until measurements stop. The gray dashed lines indicate the weekly timeframes of data assimilation and ensuing prediction; that is, the moments at which the data are measured and the forecast updated. To guide the reader, we make clear that jumps seen in the model estimate of the cell count (such as those observed at *t* = 384 h for 35 nM, or at *t* = 540 h for 75 nM) reflect model updates following assimilation of the measurements from the preceding week, resulting in a correction of the predicted cell count that had been previously over- (in the 35 nM case) or underestimated (75 nM case). The quality of our model predictions for the whole well and individual pixels resulted in *CCC*_*well*_ = .97 [.44, >.99] and *CCC*_*pixel*_ = .69 [.35, .79], respectively. Figure 8 shows a prediction performed on an individual replicate treated with 100 nM of doxorubicin. Panel 8A presents predictions of the total, surviving, and damaged populations updated every week as indicated by the vertical dashed lines (*CCC*_*well*_ = .97). Panel 8B further provides predictions of spatial dynamics and cell distribution. The top and bottom rows present the predicted cell maps and the corresponding measured data, respectively, at the time point preceding the weekly updates. After an initial phase of treatment-induced cell death and its associated global downward trend in total cell count for approximately two weeks, clusters of recovering, highly-proliferative cells become identifiable (bottom row, *t* = 384 h). These clusters remain unpredictable at this stage and can only be identified at later time points (top row, *t* > 384 h). More specifically, the prediction from week 1 (*t* = 216 h) to week 2 (*t* = 384 h) is performed by calibrating the model to the data from week 0 (*t* = *t*_*dox*_ = 48 h) to week 1 (*t* = 216 h) – when those clusters were not yet apparent (bottom row, *t* = 216 h) – and running the model forward in time. However, when predicting from week 2 to week 3 (*t* = 540 h), and therefore calibrating from week 0 to week 2, these clusters are now part of the calibrated data and the personalized prediction process enables an accurate forecast for weeks 4 and 5, where the general trend and main patterns of cell distribution and spatial growth are successfully predicted (*CCC*_*pixel*_ = .66).

**Figure 9.**
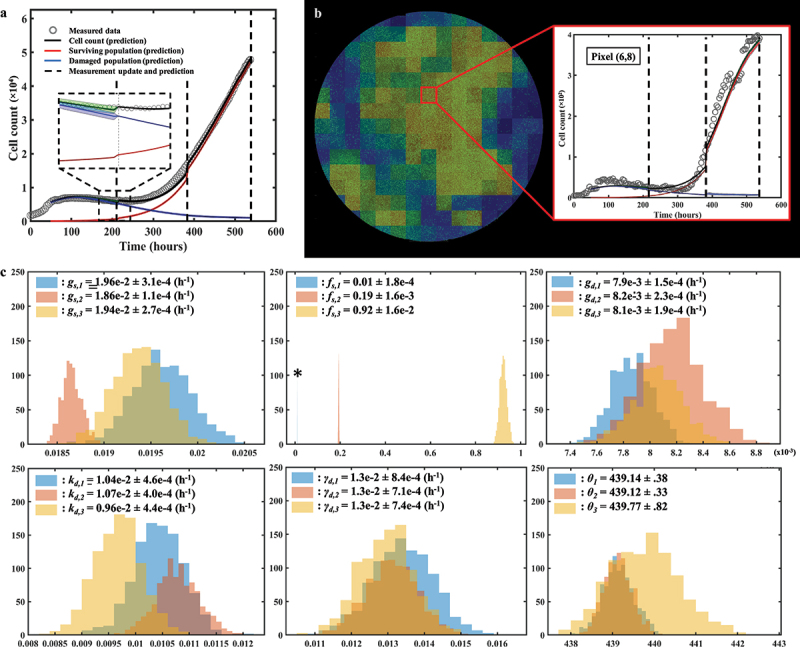
Parameter distributions of consecutive predictions following weekly data assimilation. (a) Prediction of bulk cell count on a replicate treated with 50 nM of doxorubicin. (b) Prediction of cell count on pixel (6,8). (c) Bootstrapped parameter distributions for model predictions on pixel (6,8), showing the shift in the mean value and variance of the post-treatment parameters between each weekly timeframe. The distribution for the first weekly segment of parameter *f*_*s*_ is indicated by a star as it is so narrow and may be difficult to see. The changes in the width of the parameter distributions is representative of changes in the uncertainty in the parameter value, which emanate from the quality of the model fit in the preceding weeks as well as from the wide variety of parameter values in similar pixels in the training set. The changes in the mean value point toward changes in the overall dynamics identified by the model as more data is progressively assimilated on a weekly basis.

Lastly, Figure 9 quantifies how the bootstrapped parameter distributions evolve after each weekly prediction on a replicate treated with 50 nM of doxorubicin. Panel 9A shows the bulk cell count prediction (*CCC*_*well*_ = .99), while the prediction on an individual pixel is illustrated on panel 9B. The parameter distributions used to predict each of the three weekly segments of this pixel are presented in panel 9C. In particular, we observe that *f*_*s*_ experiences a significant shift in mean and standard deviation after each week. This behavior is a consequence of the surviving population gradually outnumbering the damaged population. Moreover, the changes in the bootstrapped distributions of the proliferation rate of the surviving population (*g*_*s*_) shown in panel 9C illustrate the impact of assimilating individualized measurements of tumor cell dynamics in the model to render more accurate parameter estimations and forecasts.

When predicting the first week of data using the parameter values from the training set, the model is able to capture the usual initial growth followed by an incipient downward trend in the total tumor cell count observed after the delivery of doxorubicin in the replicates (e.g., see Figure 7). When calibrating on the first week of data (and therefore predicting the second week), the model is only exposed to the aforementioned downward trend, which translates into a narrow distribution for *g*_*s,2*_ around lower values than *g*_*s,1*_ (i.e., the assimilation of the observed cell dynamics over the first week post-treatment suggests lower surviving cell proliferation rates). However, this update to *g*_*s*_ provides a poor estimate of the dynamics of the surviving and total population over the second week because the model has not yet been informed by the sharp increase in total tumor cell counts after *t* = 300 h; Then, once the data collected during week 2 is fed to the model, the calibration provides a distribution of *g*_*s,3*_ that is similar to *g*_*s,1*_, exhibiting larger values for this parameter that match the rising trend in total tumor cell counts for this replicate. Additionally, once the tumor cell population enters a growing phase after treatment, the parameters extracted from the training set (*P*_*training*_) to calculate *g*_*s,3*_ correspond to pixels in replicates exhibiting post-therapeutic relapse as well. Since these dynamics of relapse can vary widely across the dosage levels in this dataset (see Figure 7 and ref. ^18^), the variance of the values of *g*_*s*_ corresponding to *P*_*training*_ will be large, and this translates into *g*_*s,3*_ having larger variance than *g*_*s,2*_. For *g*_*d*_, and *k*_*d*_, the predictions over the second week are once again performed by exposing the model to the downward trend of the first week. This leads to an overestimation of *g*_*d,2*_ and *k*_*d,2*_ over *g*_*d,1*_ and *k*_*d,1*_ (calibrated on the entire training set) due to the model calibration on this portion of the temporal measurements and the selection of pixels from wells with higher values for those two parameters. After assimilating the relapse of the second week, *g*_*d,3*_ and *k*_*d,3*_ are readjusted to lower values. Note that *γ*_*d*_ (the death-delay constant) experiences little shift or change in spread week after week. The rationale for this behavior is that this parameter typically influences only the first week of growth, and can thus be precisely and consistently estimated early after treatment.

## Discussion

One way to improve the treatment and care of women with breast cancer relies on a rigorous understanding of chemoresistance through the lens of tumor heterogeneity and its patient-specific nature. In this study, we have proposed a novel experimental-mathematical pipeline to model, characterize, and predict the total cell number and spatial distribution of *in vitro* colonies of MCF7 breast cancer cells over an extended time frame (up to 6 weeks). This duration is marked by an elevated amount of spatial and temporal heterogeneity generated by the cells’ treatment with doxorubicin. Our work includes an explicit separation between tumor cells surviving treatment and those irreversibly damaged, making tumor heterogeneity the main focus of our longitudinal spatiotemporally-resolved modeling and predicting effort. The addition of a spatial component to the model previously developed by Yang *et al*.^18^ represents progress in the ongoing effort to gain quantitative insights on the heterogeneous aspects of tumors which can reduce the efficacy of drugs.^4,7–9^ Utilizing a mechanism-based model offers the advantage of straightforward interpretations due to its explicit consideration of underlying biological mechanisms of (in the present case) tumor cell movement, proliferation, death due to treatment with doxorubicin and relative fractions of damaged and resistant cells. Moreover, employing a mechanism-based model provides valuable guidance for conducting additional experiments and analyses. By establishing clear relations between experimental conditions and model parameters, such studies allow identification of (for example) the effective treatment protocols and experimental designs (see, e.g., refs. ^18, 41–43^). The model was first calibrated using full time-resolved microscopy datasets yielding parameter sets that were then leveraged to forecast growth dynamics and treatment response through a data assimilation pipeline relying on a succession of short-term predictions and measurements. This approach was able to successfully recapitulate and predict the main spatiotemporal dynamics of the tumor cells following treatment with a wide range of doses of doxorubicin. A data assimilation process, as presented in this work, could be efficiently translated to clinical applications in which the microscopy data is replaced by (for example) longitudinal imaging data.^51^

Previous studies have shown the potential of mathematical oncology for predicting tumor response by developing models capturing cell growth, movement, and treatment response in both the pre-clinical and clinical settings.^18–28^ For example, Yang *et al*. investigated the interactions between glucose availability and cancer cell dynamics by leveraging a model informed by time-resolved microscopy data and consisting of a time-resolved system of ordinary differential equations.^20^ At the genomic level, Johnson *et al*. performed transcriptomic data analysis on single cells to predict treatment response through a machine learning framework incorporated into a two-phenotype ODE (ordinary differential equations)-based modeling platform.^52^ In the pre-clinical, *in vivo* setting, quantitative magnetic resonance imaging (MRI) data were acquired on murine models of glioma by Hormuth *et al*. to parametrize a reaction-diffusion model that includes angiogenesis.^53^ We sought to build on these efforts by refining the prediction approach through data assimilation, a methodology that is commonly used in weather forecasting (see ref.^54^ and implemented to predict *in vitro* glioma cell growth by Liu *et al*.^40^ Zahid *et al*. also designed prediction pipelines including a dynamic tumor carrying capacity undergoing stepwise reductions depending on the radiation dose given,^55^ or featuring adaptative dosing allowing treatment escalation and de-escalation.^56^ First initialized with population data, the models were updated with newly-collected patient-specific information to perform individual predictions of outcome. In addition, other studies have aimed at capturing heterogeneities within tumors through various approaches. Slavkova *et al*. employed a similar experimental platform as Hormuth *et al*. (quantitative MRI data of murine models) to describe the dynamics of “habitats” within gliomas subjected to radiotherapy.^31^ In our work, we have extended these studies by using a set of partial differential equations inspired by the model in ref. 18 along with a data assimilation-prediction approach adopted from ref. 40 to characterize the spatial and temporal development of MCF7 colonies over weeks.

As a novel approach to characterize spatiotemporal cell dynamics over extended timeframes, our study contains a number of limitations deserving of greater scrutiny. Firstly, we adopted a fixed spatial resolution for all the pixel cell density maps. Segmentation differences between images along with perturbations of the microscope’s field of view caused by vibrations and media changes generate noise on the signal (i.e., the cell count) of a given pixel. As such, as the grid size is made finer, the signal-to-noise ratio on a pixel basis deteriorates, leading to fitting inaccuracies and greater parameter uncertainty. While grid sizes between 3 × 3 up to 22 × 22 were investigated, the grid size of 15 × 15 used heuristically in this work allowed clear identification of the spatial patterns while limiting signal-to-noise deterioration. Moreover, given the time frame of the experiments (from 277 hours for the 10 nM dose to 963 hours for 300 nM), we decided to update the predictions on a weekly basis (168 hours). This choice was motivated by a compromise between quality of prediction and update frequency. Another approach could include, for example, updating the prediction once a specific cell count uncertainty threshold has been crossed. Lastly, while the weighting scheme for *ω* was chosen based on prior work from our team, the question of the optimal weighting scheme remains open. While other forms of weighting were investigated for this study (including a linear decrease or modifying the weights to give greater influence to *P*_*measure*_), Equation (11) was selected as it generally outperformed all other forms tried. Nevertheless, a more thorough investigation into pixel cell density generation, temporal model updating, and the weighting scheme for the training and measurement-driven parameter estimates is warranted to ensure consistency and set definitive standards on *in vitro* implementations of data assimilation techniques.

Given the number of potential free parameters in our model (i.e., Equations (1)–(7)), we had to determine if each parameter should be calibrated on either a local (i.e., one value per pixel) or a global (i.e., one value shared by all pixels across the map) fashion to reduce complexity and overfitting. To identify which parameters should be calibrated locally, a sensitivity analysis was performed and indicated that the most influential parameters on cell number were the proliferation rate of the surviving cells, *g*_*s*_, and the carrying capacity, *θ*. However, given the well-defined geometry of the experimental assay, we elected to interpret *θ* as a physical carrying capacity (i.e., the maximum number of cells that can physically fit within a fixed region of space) to be calibrated within a narrow range to further guard against potential parameter unidentifiability issues. To confirm this modeling choice other scenarios were investigated, including greater boundaries for *θ* (Figure S2 of Supplementary Materials) as well as sequentially allowing each post-treatment parameter to be calibrated locally, while calibrating the remaining five globally (Figure S3 in the Supplementary Materials). The results of these analyses indicated that the scenarios allowing *g*_*s*_ to be calibrated locally frequently outperformed the others, and that restricting *θ* to narrow range did not impact the calibration and prediction performance of our model. We also investigated scenarios in which multiple parameters were locally calibrated (Figure S2 of Supplementary Materials). These attempts unsurprisingly yielded better model calibrations, but the added complexity and parameter unidentifiability led to less accurate predictions. As obtaining the most accurate predictions was central to this study, such scenarios were discarded. We also note that it is indeed expected for the parameters controlling the dynamics of the surviving population to have a greater impact on the output than the parameters controlling the damaged population. As the damaged population is destined to die (no matter the values given to *g*_*d*_, *k*_*d*_ and *γ*_*d*_ within their boundaries), the vast majority of randomly generated scenarios in the sensitivity analysis will include a surviving population vastly outnumbering the damaged population, thus giving greater weight to the proliferation rate *g*_*s*_. While it is certainly possible to fix some of the parameters that have less impact on the model output, this would, however, impact the model’s flexibility and decrease the calibration accuracy.

As the model characterized by Equations (1)–(7) is built on biological phenomena, the calibration and prediction results can also be readily interpreted from a biological perspective. For example, as *f*_*s*_ is calibrated globally, any subset of cells at any location of the well will be divided into the same fractions of surviving and damaged cells at the time of treatment. Thus, we did not investigate specific patterns of cell distribution for the unaffected and damaged populations separately. The heterogeneity in cell count and spatial dynamics is then captured by some of the surviving cells exhibiting much higher proliferation rate than other surviving cells depending on their location (i.e., given that *g*_*s*_ = *g*_*s*_(*x*)). This approach contrasts with a calibration scheme involving, for instance, a globally-calibrated *g*_*s*_, but a locally-calibrated surviving fraction *f*_*s*_. In this scenario, the compartmentalization of surviving and damaged subpopulations at treatment onset would vary over the whole well, and hence lead to spatial heterogeneities, but post-treatment dynamics would be controlled by globally-defined parameters. As previously mentioned, the various modeling choices considered in this work were made to maximize our prediction accuracy while minimizing model complexity, and further studies are warranted to thoroughly investigate the underlying biological causes of the observed heterogeneity. We chose to calibrate diffusion in the pre-treatment phase, and then set that value to both phenotypes in the post-treatment phase. While this choice was motivated by seeking to avoid overfitting and parameter identifiability issues, we also did not have access to data that would allow us to pursue a more comprehensive parameterization of this phenomena. Additionally, our model could accommodate temporally varying parameters. As Panel 9C displays, changes in cell population dynamics between consecutive model updates can lead to difficulties predicting the different trends of treatment response observed within a single time course with temporally-fixed parameters. The appearance of highly proliferative and localized clusters of surviving cells after days or weeks of decline in the total tumor cell count points to clear changes in cellular machinery that ought to be reflected in the modeling approach. Thus, temporally varying growth rates represent a promising direction of investigation to ameliorate the model’s flexibility and better characterize such phenomena, while dynamic drug-induced death and delay rates (*k*_*d*_ and *γ*_*d*_) could characterize the various phases of drug intake and action.^18^

As both the initial cell seeding and the doxorubicin treatment are applied in a homogeneous fashion (i.e., evenly distributed across the entire well), one may hypothesize that the *in vitro* tumor cell populations would develop in a spatially-homogeneous manner. While this is observed at low (where the effects of the treatment are negligible) and high doses (where no cell recovery was observed), it is decidedly not the case within the intermediate range (i.e., 50 to 150 nM). The underlying biological causes of this heterogeneous development are not interrogated in this study and tracking more specific metrics (e.g., time-resolved live and dead cell counts, or “cell barcoding” technology for capturing phenotypic dynamics)^57,58^ could lead to greater insights on this topic. As predictions are performed and updated on a weekly basis using only cell counts and centroids positions, such phenomena may be missed by the current realization of the prediction scheme. Understanding the causes behind the rapid changes in cell number driving spatial heterogeneity (e.g., in panel B of Figure 9) would allow to capture these events more accurately. Predictions could then be further improved by anticipating changes by leveraging temporally-varying model parameters as mentioned above.

While the data employed for this study possess spatial and temporal resolution not available in the clinical setting, it allows for the development of techniques that are readily translated provided the requisite data are available. We propose that our prediction pipeline can also be implemented on clinical data available from (for example) MRI data obtained before and during treatment. Indeed, such data are already used for predicting treatment outcomes^23–29^; thus, the notion of employing a data assimilation technique accounting for spatial heterogeneity in response to systemic or local therapy is a natural next step for optimizing interventions on a patient specific basis. In particular, some radiation therapy studies do provide the opportunity for more frequent imaging during the course of therapy, thereby allowing for the implementation of a data assimilation pipeline similar to that presented in the present study.^59^ Lastly, we note that trying to predict an accurate estimate of the cell count is not necessarily required for treatment optimization in the clinical setting. For example, techniques such as functional diffusion mapping^60^ coupled with our data assimilation approach could be used to predict areas of increased proliferation, quiescence, or death to guide the delivery of radiation treatment plans.

## Conclusion

We have presented a computational-experimental platform that efficiently recapitulates and forecasts highly heterogeneous spatiotemporal dynamics of tumor cell populations before and after chemotherapy with high accuracy. In particular, our methodology can forecast the development of major spatial patterns of MCF7 cell populations after their formation following treatment with doxorubicin *in vitro*. These results represent an important step in the effort of quantifying the relationship between chemoresistance and tumor heterogeneity as well as their effect on treatment efficacy.

## Supplementary Material

Supplementary figures.docx

## Data Availability

Publicly available datasets were analyzed in his study. This data can be found here: https://doi.org/10.5281/zenodo.6973776.
