# Supplementary material for: A mathematical model for predicting the spatiotemporal response of breast cancer cells treated with doxorubicin
Source: Cancer Biol Ther. 2024 Feb 27;25(1):2321769. doi: 10.1080/15384047.2024.2321769 (PMC11057790; doi:10.1080/15384047.2024.2321769)
Supplement: Supplementary figures.docx [file KCBT_A_2321769_SM4199.docx]

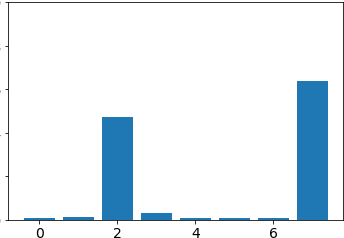


**C**

**Total effect index (*t* = 800 h)**

**1.0**

**0.8**

**0.6**

**0.4**

**0.2**

**0.0**

***Dc***

***g0***

***gs***

***fs***

***kd***

***gd***

***γd***

𝜽


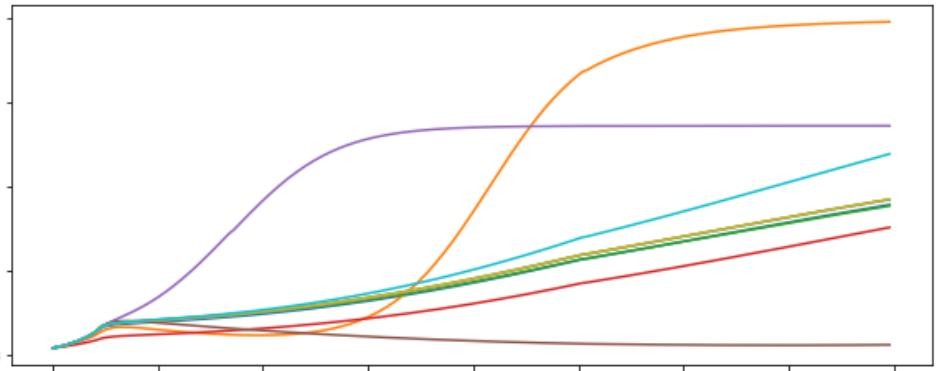


**A**

**Randomly-generated model evaluations**

**8**

**6**

**4**

**2**

**0**

**0**

**100**

**200**

**300**

**400**

**500**

**600**

**700**

**800**

**Time (hours)**


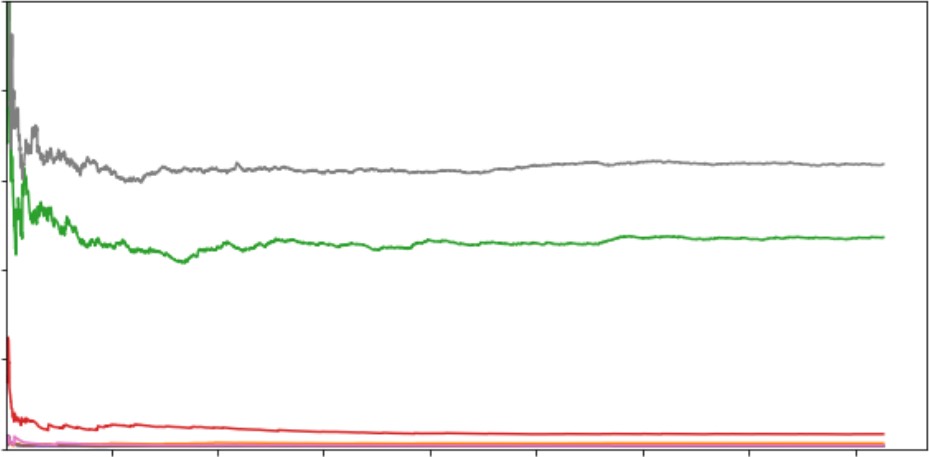


**B**

**Total effect index convergence**

**1.0**

**0.8**

***Dc g0***

***gs fs***

***kd gd***

***γd***

𝜽

**0.6**

**0.4**

**0.2**

**0**

**0**

**0.5**

**1**

**1.5**

**2**

**2.5**

**3**

**3.5**

**4**

**Samples (×103)**

**Total effect index**

**Cell count (x104)**

**Total effect index**

**Figure S1 : Sensitivity analysis of two species phenotypic model. A.** Examples of randomly generated model evaluations**.** The model was evaluated 5000 times over a timeframe of 800 hours by randomly sampling parameters within their respective boundaries along with initial conditions similar to those observed experimentally (model evaluations with cell counts diverging because of numerical errors were discarded). **B**. Convergence on total effect indices with respect to number of samples. **C**. Results of sensitivity analysis. As shown, the parameters with the greatest influence on the cell count are the growth rate of the surviving population *g_s_* and the carrying capacity 𝜃.


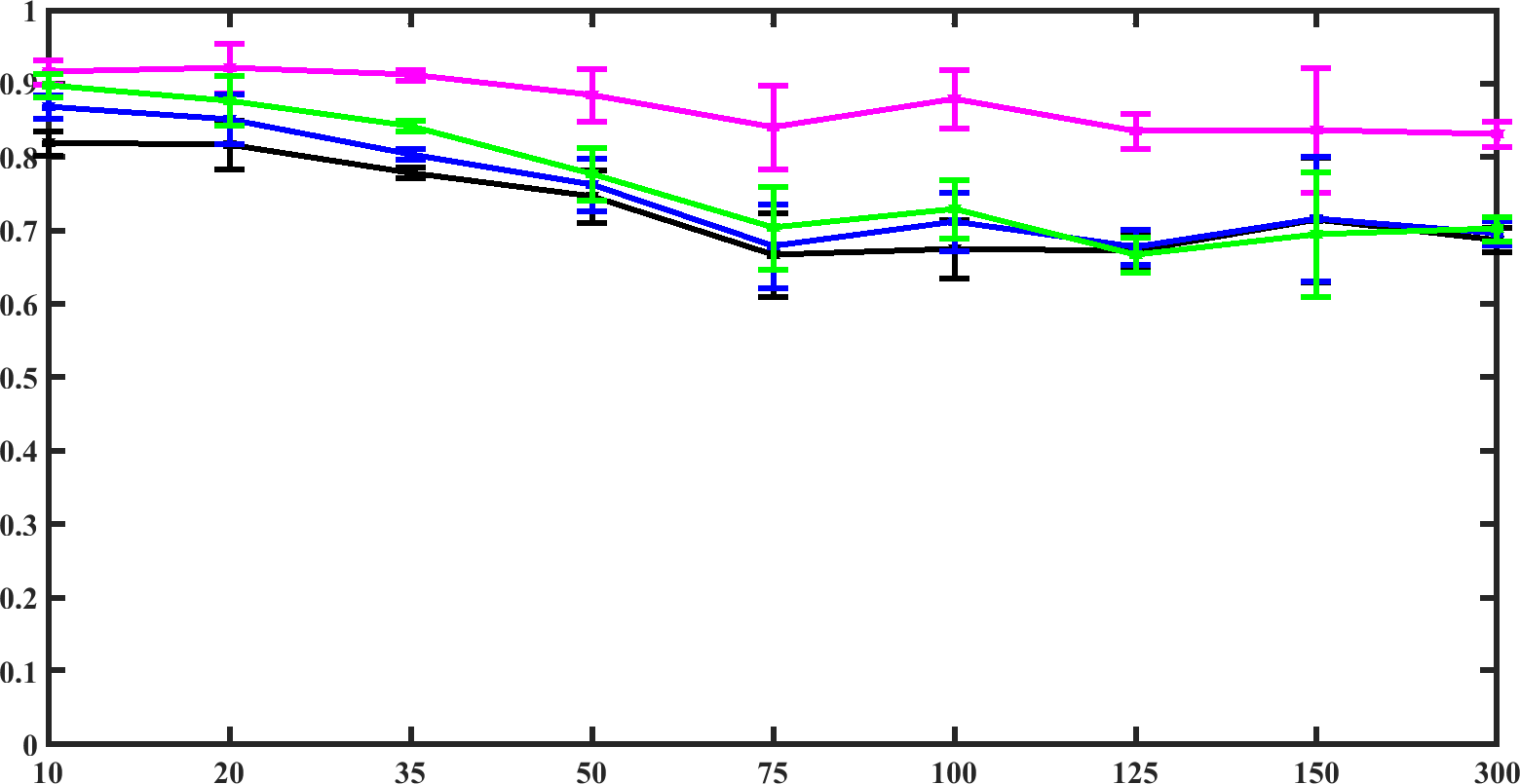


**B**

**Calibration results**

**Scenario 1**

**Scenario 2**

**Scenario 3**

**Scenario 4**

**Dosage (nM)**

***CCCpixel***

***CCCpixel***

**A**

**Summary of modeling scenarios**

**Scenario 1**

- **Local** 𝒈_𝒔_
- **All other parameters**

**global**

- 𝜽 **between 88000- 90000**

**Scenario 2**

- **Local** 𝒈_𝒔_
- **All other parameters**

**global**

- 𝜽 **between 15000-**

**90000**

**Scenario 3**

- **Local** 𝒈_𝒔_ **and** 𝜽
- **All other parameters global**
- 𝜽 **between 15000-**

**90000**

**Scenario 4**

- **All parameters local**
- 𝜽 **between 15000-**

**90000**

**Figure S2 : Calibration and prediction results for alternative model parameterization scenarios. A.** Four scenarios were investigated to validate the modeling decisions made in this study. **B.** Calibration results for all dosages and the four scenarios. Solid lines correspond to the mean of the 6 replicates receiving each dosage of doxorubicin (error bars : standard deviation). Calibrating all the parameters locally provides the best calibrations. **C.** Prediction results for all dosages and the four scenarios. Solid lines correspond to the mean of the 6 replicates (error bars : standard deviation). Due to increased complexity and parameter unidentifiability, calibrating all the parameters locally underperforms with respect to the other scenarios when making predictions. Notice that poorer performance is especially observed for the dosages for which higher spatial heterogeneity is observed in the experimental data


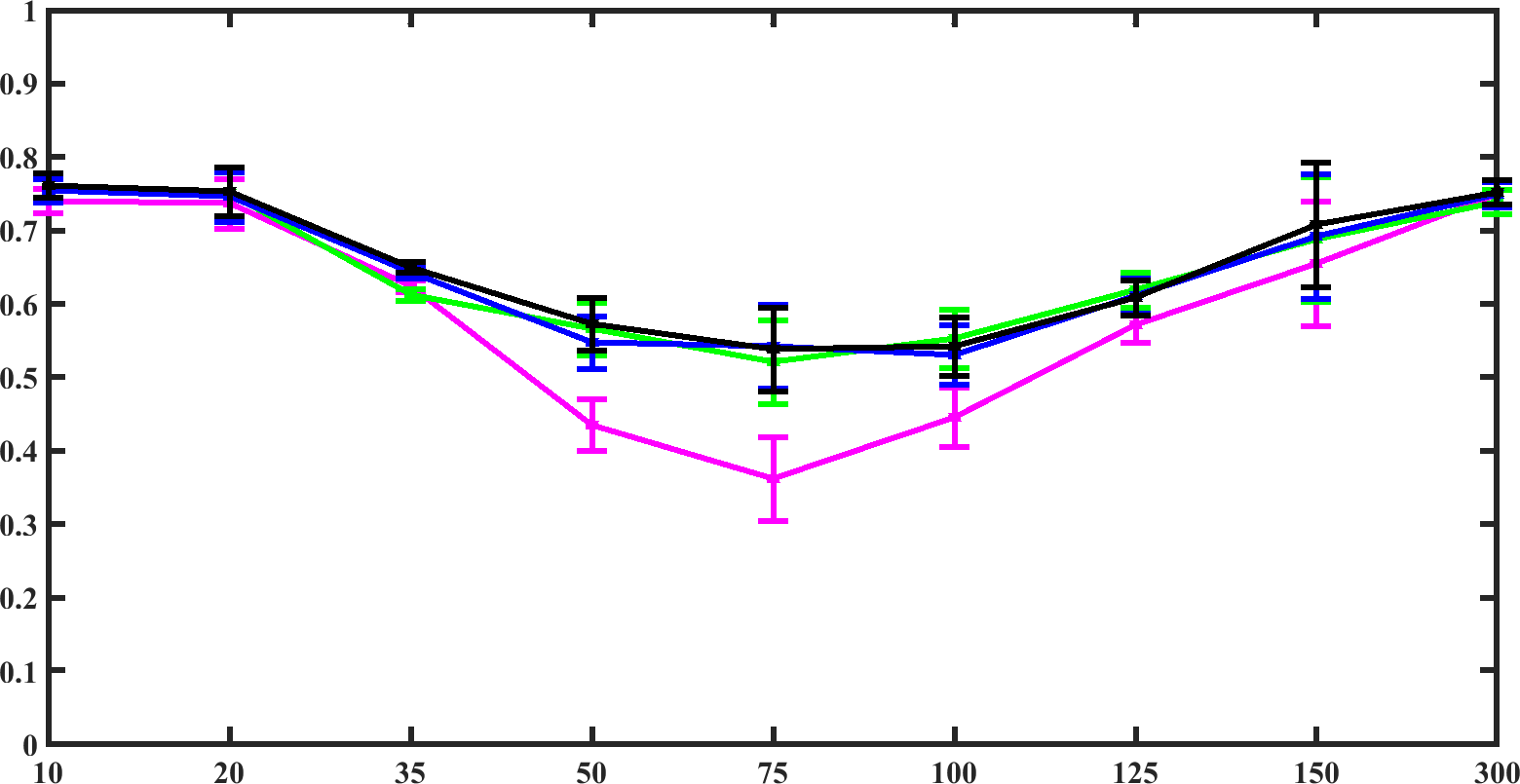


**C**

**Prediction results**

**Scenario 1**

**Scenario 2**

**Scenario 3**

**Scenario 4**

**Dosage (nM)**


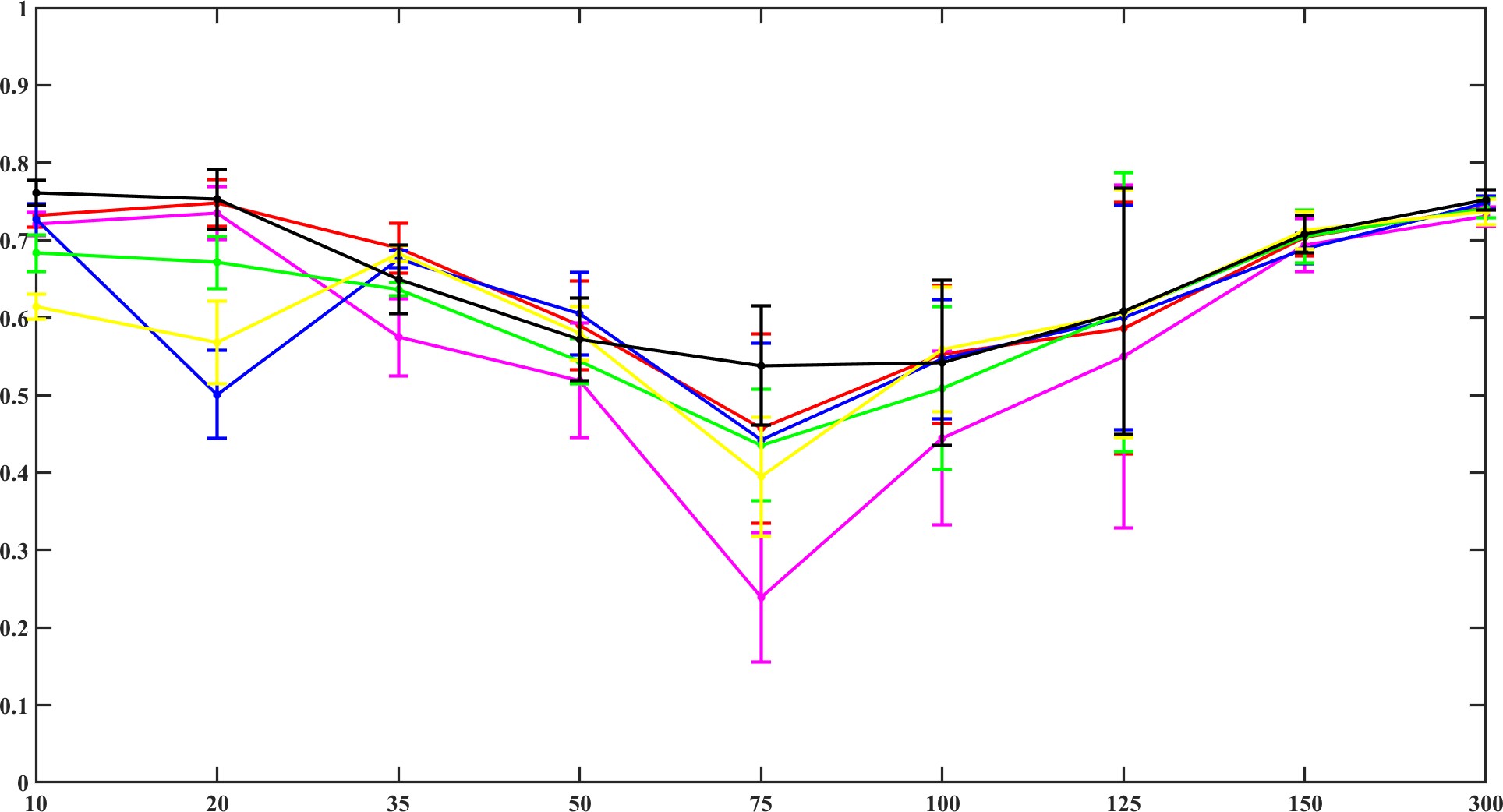


**Prediction results**

**Local *g_s_* Local *f_s_* Local *k_d_* Local *g_d_* Local *γ_d_* Local** 𝜽

**Dosage (nM)**

***CCCpixel***

**Figure S3 : Prediction results for alternative choices of a single locally calibrated parameter.** In this figure, we consider six alternative model parameterizations within our data assimilation-prediction pipeline, such that only one of the six post-treatment parameters *(g_s_, f_s_, k_d_, g_d_, γ_d_,* 𝜃) was calibrated locally while the other five were calibrated globally. The scenario where the surviving proliferation rate *g_s_* is calibrated locally performs either equally or better than the other scenarios, validating the results of sensitivity analysis in this study.
